# Inhibition of glucose assimilation in *Auxenochlorella protothecoides* by light

**DOI:** 10.1186/s13068-020-01787-9

**Published:** 2020-08-18

**Authors:** Yibo Xiao, Jianying Guo, Huachang Zhu, Anwar Muhammad, Haiteng Deng, Zhangli Hu, Qingyu Wu

**Affiliations:** 1 Guangdong Technology Research Center for Marine Algal Bioengineering, Guangdong Key Laboratory of Plant Epigenetics, College of Life Sciences and Oceanography, Shenzhen University, Shenzhen, 518060 People’s Republic of China; 2 Key Laboratory of Optoelectronic Devices and Systems of the Ministry of Education and Guangdong Province, College of Optoelectronic Engineering, Shenzhen University, Shenzhen, 518060 People’s Republic of China; 3 Key Laboratory of Industrial Biocatalysis of the Ministry of Education and Center for Synthetic and Systems Biology, School of Life Sciences, Tsinghua University, Beijing, 100084 People’s Republic of China; 4 Shenzhen Key Laboratory of Marine Bioresource and Eco-Environmental Science, Longhua Innovation Institute for Biotechnology, Shenzhen University, Shenzhen, 518060 People’s Republic of China

**Keywords:** Microalgae, Heterotrophic cultivation, Glucose assimilation, Biomass, Comparative proteomics

## Abstract

**Background:**

The yield of microalgae biomass is the key to affect the accumulation of fatty acids. A few microalgae can assimilate organic carbon to improve biomass yield. In mixotrophic cultivation, microalgae can use organic carbon source and light energy simultaneously. The preference of the main energy source by microalgae determines the biomass yield. *Auxenochlorella protothecoides* is an oleaginous mixotrophic microalga that can efficiently assimilate glucose and accumulate a large amount of biomass and fatty acids. The current study focused on the effect of light on the growth and glucose assimilation of *A. protothecoides*.

**Results:**

In this study, we found that the uptake and metabolism of glucose in *A. protothecoides* could be inhibited by light, resulting in a reduction of biomass growth and lipid accumulation. We employed comparative proteomics to study the influence of light on the regulation of glucose assimilation in *A. protothecoides*. Proteomics revealed that proteins involving in gene translation and photosynthesis system were up-regulated in the light, such as ribulose-phosphate 3-epimerase and phosphoribulokinase. Calvin cycle-related proteins were also up-regulated, suggesting that light may inhibit glucose metabolism by enhancing the production of glyceraldehyde-3-phosphate (G3P) in the Calvin cycle. In addition, the redox homeostasis-related proteins such as thioredoxin reductase were up-regulated in the light, indicating that light may regulate glucose uptake by changing the redox balance. Moreover, the increase of NADH levels and redox potential of the medium under illumination might inhibit the activity of the glucose transport system and subsequently reduce glucose uptake.

**Conclusions:**

A theoretical model of how glucose assimilation in *A. protothecoides* is negatively influenced by light was proposed, which will facilitate further studies on the complex mechanisms underlying the transition from autotrophy to heterotrophy for improving biomass accumulation.

## Background

Most microalgae are photoautotrophic microorganisms that need light as an energy source and carbon dioxide as carbon source to ultimately synthesize valuable products, such as polyunsaturated fatty acids (PUFAs), carotenoids, proteins, etc. [1]. Nevertheless, several microalgal species are capable of growing heterotrophically by assimilating sugars, exhibiting metabolic adaptability and flexibility. Prominent examples of such mixotrophic species include *Prochlorococcus* sp. [1], *Auxenochlorella protothecoides* (Synonym as *Chlorella protothecoides*) [2, 3], *Chlorococcum* sp. GD [4], and *Chlorella zofingiensis* [5]. The yield of value-added metabolites from microalgae depends on the intracellular content of metabolites and the biomass yield. Consequently, increasing biomass accumulation is a central strategy for increasing the yield of target products. The cultivation of mixotrophic microalgae in heterotrophic mode can increase the concentration of biomass by as much as 25-fold compared with photoautotrophic cultivation [6]. Moreover, mixotrophic organisms are capable of photosynthesizing and assimilating organic carbon, which also significantly increases biomass productivity [7]. Genetic engineering of mixotrophs to improve their utilization of exogenous glucose by introducing heterologous glucose transporters was achieved in *Phaeodactylum tricornutum* [8] and *Synechococcus elongatus* PCC 7942 [9]. The utilization of sugars is essential for the heterotrophic growth mode of mixotrophic microalgae. The oxidation of sugars in heterotrophic and mixotrophic metabolic modes provides an increased supply of energy and metabolic precursors, allowing for faster cell growth and continuous production of target chemicals.

*Auxenochlorella protothecoides* is a typical oleaginous microalga with a great potential application value in the production of biodiesel and lutein [10–12]. In the heterotrophic mode under dark and nitrogen-depleted conditions, *A. protothecoides* can use glucose efficiently by utilizing at least nine glucose transporters [13]. *A. protothecoides* can transition between heterotrophy and photoautotrophy in response to light/dark and the C/N ratio changes [14], and, thus, has multiple metabolic pathways that can be adapted for the manufacture of valuable products. Previous reports indicated that mixotrophic cultivation of *A. protothecoides* allows for higher biomass production than heterotrophic cultivation [15, 16]. Under appropriate conditions of illumination and nutrient availability, *A. protothecoides* can also grow mixotrophically, simultaneously utilizing photosynthesis and heterotrophy. Light plays an important role in the regulation of enzymes involved in photosynthesis and is also the critical signal for the switch from heterotrophic to autotrophic growth in microalgae or in the post-germinative development of higher plants [17–20]. Conversely, the availability of glucose was found to affect photosynthesis in mixotrophs. Accordingly, high concentrations of glucose (6–10 g/L) not only improved biomass accumulation but also inhibited photosynthesis [4, 10]. However, the effect of photosynthesis on glucose utilization in microalgae is understudied.

In this study, we aimed to gain insights into the influence of light on glucose uptake and metabolism. Therefore, a comparative 2D LC–MS/MS analysis was performed to reveal the differences in the protein expression profile between *A. protothecoides* cultured in the presence of glucose under light and dark conditions. This comparison allowed us to identify the differentially abundant proteins involved in glucose metabolism and photosynthesis. Additionally, the redox state of the cells is known to be an important signal in higher plants. For example, the activity of the SUT1 protein from potato (*Solanum tuberosum*, *St*SUT1) is regulated by redox conditions during the day/night (light/dark) cycles which regulate the circadian clock [21]. We compared the redox state inside and outside of cells under light and dark conditions. Taken together, this study provides new insights into the influence of light on glucose transport and metabolism. Finally, we developed a tentative model of how light regulates glucose assimilation, which will facilitate further study on the heterotrophic transition in microalgae for enhancing biomass accumulation.

## Results and discussion

### The differences in glucose assimilation under light and dark conditions

**Fig. 1:**
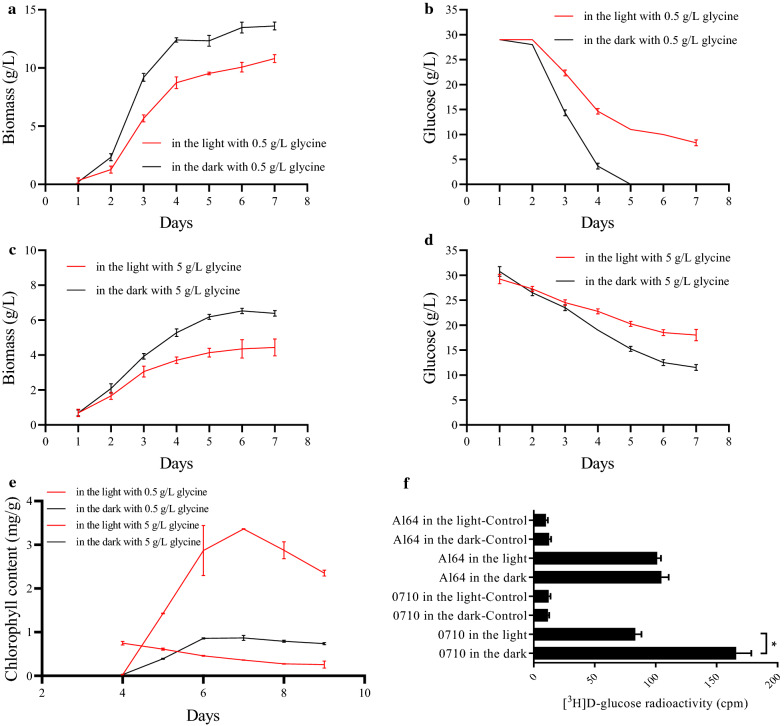
Time curves of growth, glucose consumption and glucose transport ability under different culture conditions. **a** Biomass accumulation under the light and dark conditions with 0.5-g/L glycine; **b** concentration of residual glucose under the light and dark conditions with 0.5-g/L glycine; **c** biomass accumulation under the light and dark conditions with 5-g/L glycine; **d** concentration of residual glucose under the light and dark conditions with 5-g/L glycine; **e** chlorophyll content under the light and dark conditions with 0.5- and 5-g/L glycine; **f** comparison of the glucose transport ability of *A. protothecoides* 0710 and the photosynthesis-deficient mutant Al64 under light and dark conditions. Glucose uptake by microalgae cells was assayed at 4 days of cultivation in the light or dark. Microalgae cells without adding d-[2-^3^H] glucose was used as a negative control (in the light control/ in the dark control)

The *A. protothecoides* was cultured in a 100-mL shake flask using conventional nitrogen-limited heterotrophic medium. The experimental group was placed under light (mixotrophic culture—MC) and the control group was placed in the dark (heterotrophic culture—HC). The biomass and residual glucose concentration in the medium were measured to compare the growth and glucose consumption of the cultures. As shown in Fig. 1a, the growth of the MC was slower than that of the HC, which depleted glucose on the 5th day. By contrast, glucose was not exhausted on the 7th day in the MC (Fig. 1b). The residual glucose concentration in the MC was higher than in the HC, indicating that *A. protothecoides* assimilated less glucose in the light. Many microalgae, such as *Phaeodactylum tricornutum*, *Chlorella vulgaris* and *Chlorococcum* sp. GD, can use glucose when grown in light [22, 23]. When such organisms are grown in illuminated mixotrophic culture, the biomass will be higher due to the concomitant activity of both photosynthesis and organic carbon assimilation. Therefore, the phenotype of *A. protothecoides* of reduced growth and glucose assimilation in the light is different from other microalgae. The specific cell growth rates of *A. protothecoides* cells in autotrophic and heterotrophic mode were found to be 0.0028 and 0.0257 h^−1^, respectively [24]. The specific cell growth rate in heterotrophic mode was 9 times higher than in autotrophic mode, indicating that carbon fixation via photosynthesis contributed less to the increase of biomass when both light and glucose were present.

Nitrogen is an essential element for chlorophyll and protein synthesis. Consequently, a sufficient supply of nitrogen sources can promote photosynthesis in microalgae [25]. After increasing the concentration of the nitrogen source to a high level (e.g., 5 g/L glycine), the growth rate and glucose consumption were also reduced under illumination (Fig. 1c, d). The chlorophyll content was highest in the light with 5-g/L glycine, while the cells contained almost no chlorophyll in the dark with 0.5-g/L glycine (Fig. 1e). Furthermore, cells grown in the dark with 5-g/L glycine and in the light with 0.5-g/L glycine had low levels of chlorophyll. The chlorophyll content under high nitrogen source concentrations was significantly higher than under low nitrogen, but the growth rate and glucose consumption observed at the high nitrogen source concentration was also inhibited by light. Therefore, the reduced accumulation of biomass and inhibition of glucose consumption in the light was not simply caused by the photosynthetic fixation of carbon dioxide.

*Auxenochlorella protothecoides* can efficiently import glucose from the medium via a specific glucose transporter system. Gao et al. identified nine transporter homologs in *A. protothecoides* using the HUPs (H^+^/hexose co-transporter) from *Chlorella kessleri* as the query sequence [13]. Moreover, three of the identified HUP-like proteins might be responsible for the ability of *A. protothecoides* to rapidly import glucose. Radiolabelled substrate can be used to measure the rate of sugar transport and the ability of sugar uptake more accurately. The sucrose transporter StSUT1 from potato (*Solanum tuberosum*) was expressed in yeast and the transport activity and post-translational modification of StSUT1 were measured using ^14^C-Sucrose [21]. Unlike the tissues of higher plants, the unicellular *A. protothecoides* can be directly used to measure the glucose transport rate in vivo. *A. protothecoides* Al64 is an autotrophy-deficient strain obtained by ethyl methanesulfonate mutagenesis. The glucose transport activity of wild-type *A. protothecoides* and Al64 was measured using radiolabelled d-[2-^3^H] glucose. In Fig. 1f, the X coordinate represents the radioactivity generated by d-[2-^3^H] glucose, which had been transported into cells within 3 min. This value reflects the glucose transport rate. The results showed that the glucose transport rate of wild-type cells in the dark was almost two times higher than under illumination (*p* = 0.013 < 0.05), indicating that light could inhibit the glucose transport. By contrast, there was no significant difference in Al64 under the light and dark conditions. Hence, the glucose transport activity of Al64 was not affected by light, indicating that the inhibition of the glucose transport activity of *A. protothecoides* by light is related to the photosynthesis system and may be adjusted by changes in the intracellular environment.

**Fig. 2:**
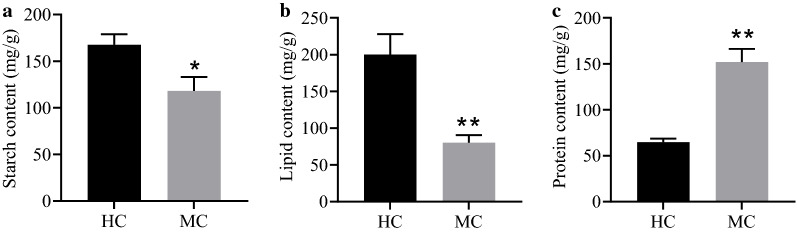
Cell components of *A. protothecoides* from heterotrophic culture (HC) and mixotrophic culture (MC). **a** Starch content of *A. protothecoides*. **b** Lipid content of *A. protothecoides*. **c** Protein content of *A. protothecoides*

The lipid content of *A. protothecoides* cells grown in heterotrophic mode four times higher (55.20%) than in the autotrophic mode [12]. The heterotrophically grown cells were filled with large lipid droplets, while the lipid droplets in the autotrophically grown cells were smaller and fewer [26]. Subsequently, the differences of subcellular structure between cells from the MC and the HC were observed by TEM (Additional file 1: Figure S1). The structure of chloroplasts was not found in cells from the HC, while incomplete chloroplast structure was observed in cells from the MC, indicating that the structure of chloroplast was still retained under nitrogen limitation condition combined with illumination. The efficiency of PSII (ФPSII) of *A. protothecoides* in the MC and HC was detected (Additional file 1: Figure S2). The results suggested ФPSII of *A. protothecoides* was rarely determined in the dark. Notably, ФPSII of MC was increased after 4 days of cultivation. It is suggested that incomplete chloroplast structures of *A. protothecoides* still have photosynthesis ability. The diameter of lipid droplet was larger than 2 μm in cells from the HC and smaller than 2 μm in those from the MC. The differences of starch, lipid and protein contents between cells from the HC and the MC were measured (Fig. 2). The starch and lipid contents of the MC were lower than that of the HC, indicating that lipid accumulation was inhibited under illumination (Fig. 2a, b). The protein contents of the MC were significantly higher than that of the HC (*p* < 0.05) (Fig. 2c). This may be due to the photosynthetic system requiring more proteins in the MC than HC. Under nitrogen limitation, most of the precursors for lipid biosynthesis were derived from extracellular glucose. The difference between lipid and protein accumulation between the HC and MC indicated that light also influenced glucose metabolism.

### Comparative proteomic analysis of A. protothecoides cells grown under illumination and in the dark

Comparative proteomics was used to study the mechanism by which light regulates glucose uptake in *A. protothecoides*. Protein samples were labeled with TMT, identified by tandem mass spectrometry, and analyzed using the *A. protothecoides* protein database. A total of 4820 proteins were identified in the samples, accounting for 68.5% of the total known proteins of *A. protothecoides*. The proteins with ratios greater than 1.3 or lower than 0.77 and *p-*values of less than 0.05 were identified as the differentially expressed proteins (DEPs) between the HC and MC. A total of 240 up- and 98 downregulated proteins were identified (Additional file 2: Table S1, S2).

**Fig. 3:**
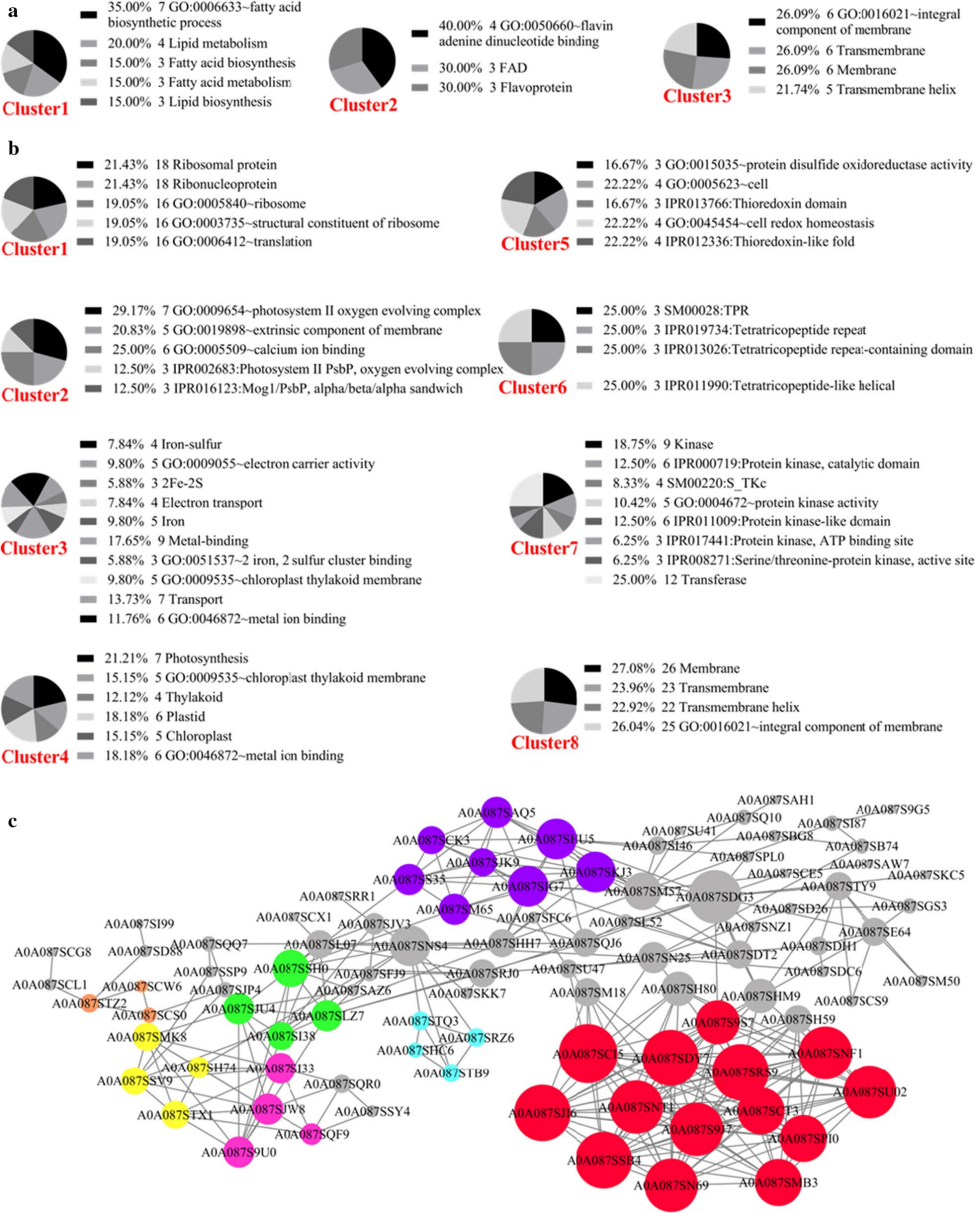
Clustering of DEPs between MC cells and HC cells. **a** DAVID clustering of downregulated DEPs; **b** DAVID clustering of upregulated DEPs; **c** protein–protein interaction network of DEPs

The online functional annotation tool DAVID was used to analyze the up- and downregulated proteins separately [27]. The downregulated proteins were grouped into 3 clusters (Fig. 3a). The proteins in Cluster1 (Enrichment Score: 3.45) were mainly related to the biosynthesis and metabolism of lipids and fatty acids, which was consistent with the observed smaller lipid droplets in the cells from the MC. The proteins in Cluster2 (Enrichment Score: 1.67) were identified as having a non-covalent interaction with flavin adenine dinucleotide (FAD), which is also involved in the metabolism of pyruvic acid, fatty acids, oxidative degradation of amino acids and the electron transport chain. Cluster3 (Enrichment Score: 0.004) included integral membrane proteins and transmembrane proteins.

The upregulated proteins were grouped into 8 clusters (Fig. 3b). Cluster1 (Enrichment Score: 4.58) contained many ribosomal proteins involved in the protein translation process. The upregulation of ribosomal proteins might be related to the increased requirements for proteins due to the construction of photosynthesis systems in the MC. The proteins of Cluster2 (Enrichment Score: 2.98) were associated with the photosynthesis system. It was shown that even under nitrogen-limited conditions (glycine concentration = 0.5 g/L), light could still promote the construction of the photosynthesis system. Cluster3 (Enrichment Score: 1.26) included iron–sulfur proteins and metal-binding proteins. Most proteins in this cluster were related to the electron transport chain of photosynthesis, indicating that the photosynthetic electron transport chain became active. Cluster4 (Enrichment Score: 1.2) was also related to photosynthesis. Importantly, all of the four clusters were consistent with the observed phenotypes, indicating that *A. protothecoides* needs to synthesize more proteins in MC to construct the photosynthetic system. Cluster 5 (Enrichment Score: 0.62) included proteins with sulfide oxidoreductase activity and those involved in intracellular redox balance. Thiol–disulfide exchange reduction/oxidation (redox) reactions regulate carbon fixation, respiration, and metabolic processes in plant cells [28]. The redox system plays a key role in maintaining cellular homeostasis and regulating cell growth and metabolism [29]. Under illumination, the redox homeostasis-related proteins in *A. protothecoides* were upregulated, indicating a change in intracellular redox balance. Hence, the redox balance was changed by light, and the change of cell homeostasis provided new clues for studying the influence of light on glucose assimilation. The enrichment scores of clusters 6, 7, and 8 were, respectively, only 0.37, 0.35 and 0.15, indicating that the enrichment of protein functions in these three clusters was poor.

Since *A. protothecoides* and *Chlamydomonas reinhardtii* are closely related, the protein–protein interaction (PPI) database of *C. reinhardtii* from STRING was used to analyze the interaction relationships of DEPs of *A. protothecoides* based on homology alignment [30]. The DEPs in the PPI network numbered in accordance with the accession numbers of the proteins in the UniProt database (Fig. 3c). The different colors indicate that they were clustered according to similar functions or compositions. The red cluster contained the ribosome-associated proteins. The purple cluster was involved in carbon metabolism, indicating that light had an effect on carbon metabolism. Most proteins in the blue cluster were oxidoreductases. Proteins in the green cluster were enzymes associated with the tricarboxylic acid cycle. The purple–red cluster contained proteins involved in the synthesis of lipids. A yellow cluster was associated with fatty acid metabolism. Proteins in the orange cluster were associated with chlorophyll synthesis. Importantly, the results of PPI network analysis were consistent with the results of DAVID analysis.

The DEPs indicated by gray circles were not clustered. The largest gray circle was A0A087SDG3, which had the most interactions with other ungrouped proteins. A0A087SDG3 was predicted to be a thioredoxin reductase, and it was upregulated. Thioredoxin reductase, thioredoxin and NADPH constitute the thioredoxin system, which plays an important role in maintaining the intracellular redox balance. Although thioredoxin reductase was not clustered in the PPI network, the number of interacting proteins reflects a wide range of effects on DEPs. Combined with the analysis of the upregulated proteins in Cluster5, the DEPs and PPI information all reflected the significant changes in the expression of proteins related to the redox balance under illumination. This suggested that the intracellular redox balance was affected by light and this effect was likely related to the observed regulation of glucose assimilation.

### Pathway mapping of DEPs in KEGG

The DEPs were uploaded to KEGG Mapper for pathway enrichment, whereby the upregulated proteins were marked in red and the downregulated proteins were marked in blue [31]. The annotated proteins in the *A. protothecoides* genome were marked in green. According to the phenotype of influencing glucose assimilation under illumination, KEGG pathway analysis focused on photosynthesis, glycolysis, fatty acid biosynthesis and carbon fixation.

There were obvious differences in the subcellular structure of cells from the MC and the HC. Chloroplastic structures were apparent in the cells form the MC. While these were not as distinct as in the autotrophically grown cells, they nevertheless indicated that there was active photosynthesis in the MC (Fig. 2). All proteins enriched in the photosynthesis pathway were upregulated, including subunits of photosystem I (PSI), photosystem II (PSII) and the cytochrome b6-f complex (Additional file 1: Figure S3). All top forty proteins were almost related to photosynthesis, indicating that light could promote the construction of the photosynthetic systems even under nitrogen-limited culture conditions. In addition to the constituent proteins of the photosystem reaction center, there were three oxygen-evolving enhancer proteins (OEE) among the top eight upregulated proteins. The upregulation fold changes of PsbO (OEE1), PsbP (OEE2) and PsbQ (OEE3) reached 8.09, 6.96, and 6.21, respectively. OEE is an important component of the oxygen-evolving complex (OEC) in PSII, which together with manganese clusters participates in the water splitting reaction to generate oxygen and electrons. Under NaCl- and heat-stress, the extended structure of OEE can protect the reaction center D1 protein from oxygen radicals [32, 33]. Forster et al. found that two very high light (VHL)-resistant mutants of *C. reinhardtii* upregulated OEE1 expression under high light stress [34]. OEE was also found to have a thioredoxin-like activity in *Chlorella* [35]. Three OEE proteins were highly upregulated in MC, which could improve the function of intracellular OEC to maintain the activity of PSII and redox balance. Under nitrogen-limited conditions, the coexistence of glucose and light may be a stress factor for *A. protothecoides* cells. In response to stress, intracellular metabolism undergoes a series of adjustments that alter the accumulation of metabolites.

**Fig. 4:**
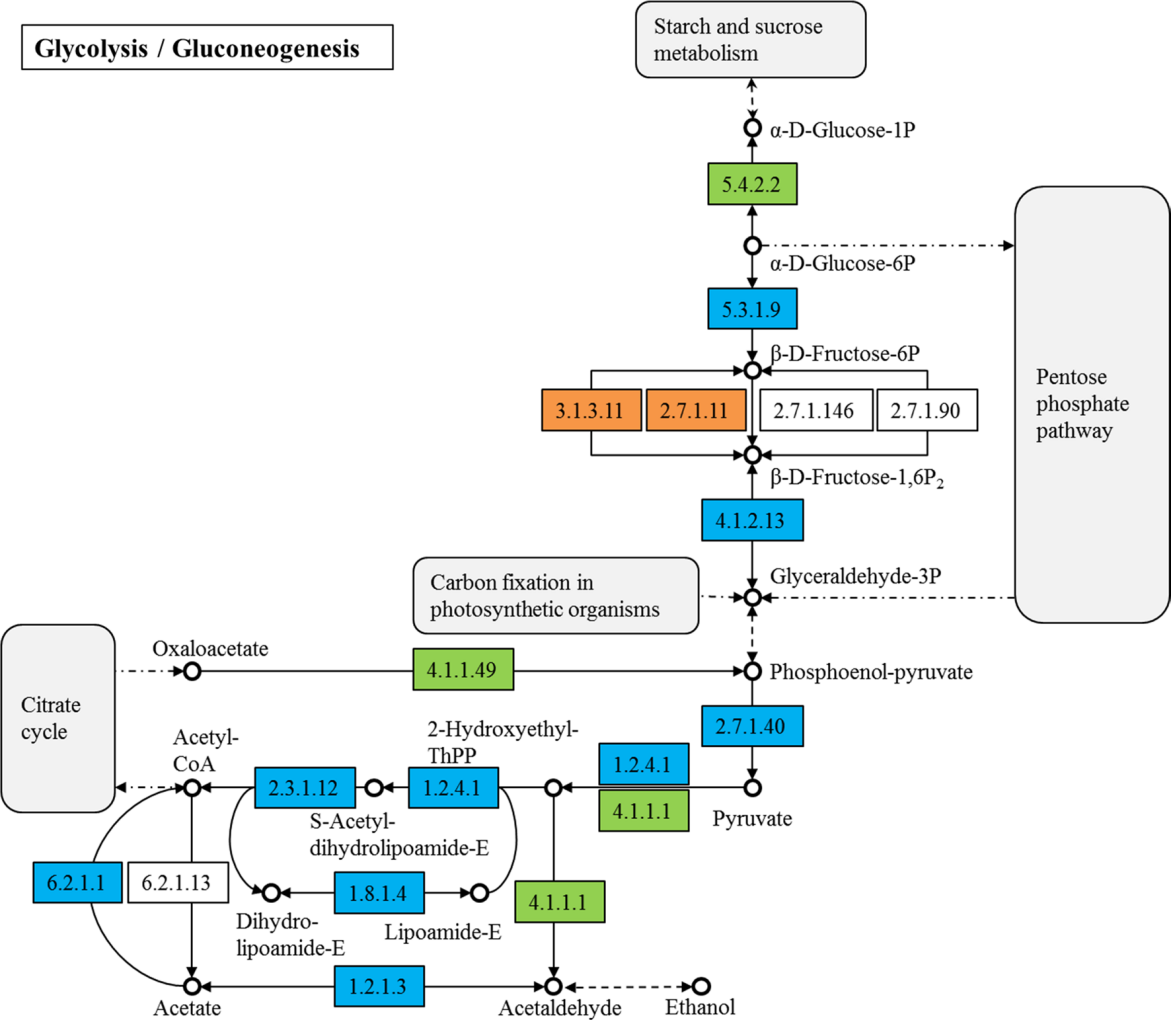
DEPs in glucose metabolism

Light has an influence on glucose assimilation, including glucose transport and metabolism. Exogenous glucose first enters the glycolysis pathway to produce pyruvate (Fig. 4). Most of the DEPs in the glycolysis pathway were downregulated. Two enzymes that catalyze the production of d-glyceraldehyde 3-phosphate from d-glucose 6-phosphate, as well as pyruvate kinase were downregulated, which may eventually reduce the synthesis of pyruvate. The enzymes that are responsible for the producing acetyl-CoA from pyruvate, i.e., pyruvate dehydrogenase, dihydrolipoic acid dehydrogenase, dihydrothionyl dehydrogenase, acetaldehyde dehydrogenase, and acetyl-CoA synthase were also downregulated (Fig. 4), indicating a decrease in the synthesis of acetyl-CoA. Most of the enzymes that are involved in the fatty acid biosynthesis pathway were downregulated (Additional file 1: Figure S4). Acetyl-CoA is a precursor for fatty acid biosynthesis, and reduction of the biosynthesis of acetyl-CoA may be responsible for the decrease of fatty acid biosynthesis. The downregulation fold changes of citrate synthase and dihydrolipoyl dehydrogenase in TCA cycle were 0.75 and 0.66. Malate dehydrogenase was upregulated slightly. The downregulation of citrate synthase, which controls the initiation of TCA cycle, suggested a weakening of the TCA cycle, which might be one of the reasons for the reduced growth rate under illumination (Additional file 1: Figure S5).

**Fig. 5:**
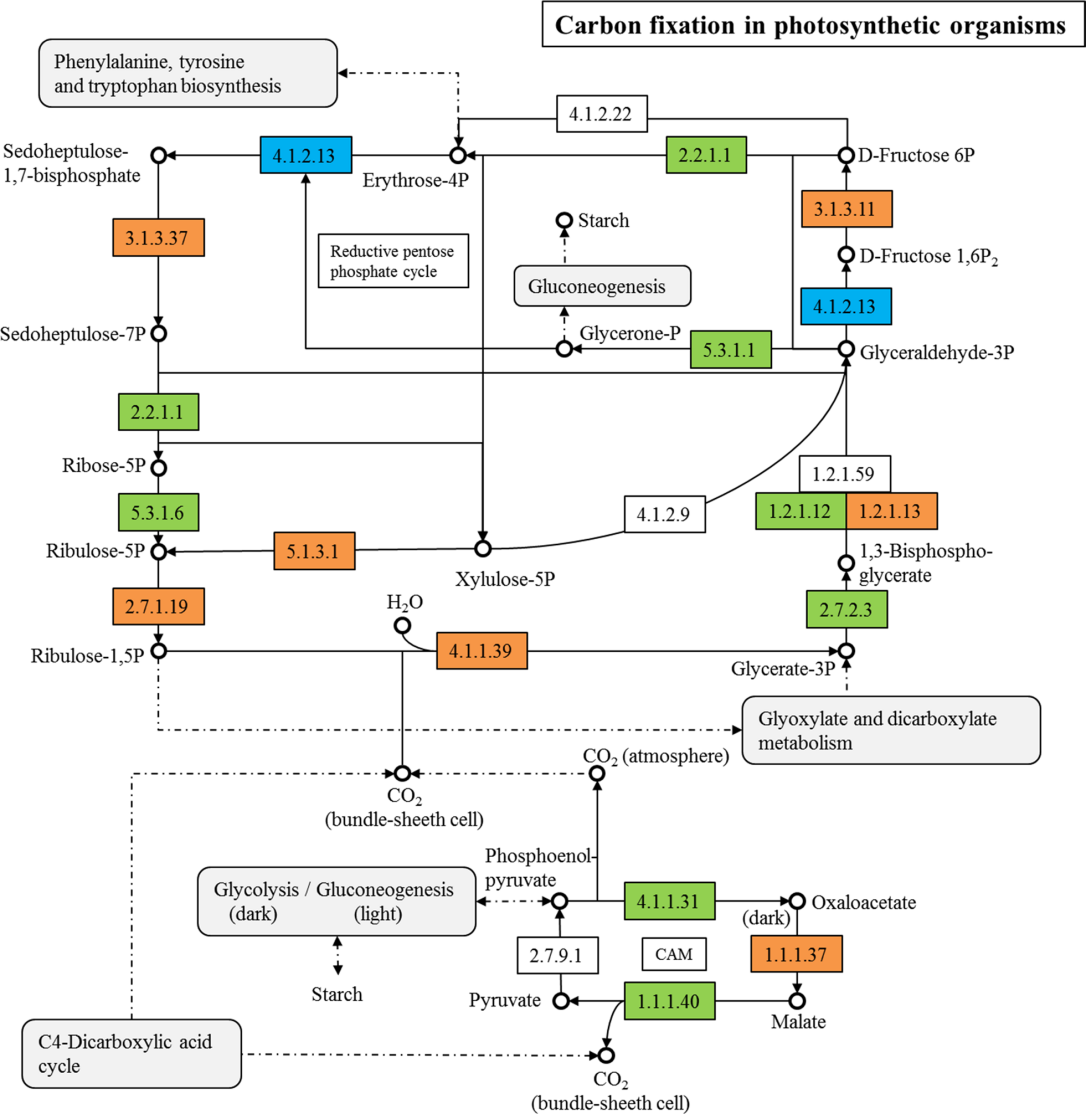
DEPs in carbon fixation

The results showed that ribulose-phosphate 3-epimerase and phosphoribulokinase, which catalyze the production of ribulose bisphosphate (RuBP) in the carbon fixation pathway of photosynthetic organisms, were upregulated 1.55 and 3.56 times, respectively (Fig. 5). The RuBP carboxylase and glyceraldehyde-3-phosphate dehydrogenase in the Calvin cycle were upregulated 2.78 and 2.62 times, respectively, indicating that the synthesis of RuBP was increased and the Calvin cycle was also enhanced. In the Calvin cycle, CO_2_ is converted into G3P, which is also an intermediate product of glycolysis. Under illumination, the G3P produced by the Calvin cycle may promote the reverse reaction of fructose-bisphosphate aldolase in glycolysis, thereby inhibiting glucose metabolism. Therefore, light might inhibit the glucose metabolism via chemical products of the dark reaction of photosynthesis.

### Redox analysis of A. protothecoides under light and dark conditions

Previous studies have shown that higher plants also undergo redox changes during the night and day, thereby regulating the activity of sucrose transporters [36]. Redox states integrate and coordinate the energy production and status within a cell to regulate homeostasis and metabolism [37]. The results obtained from the DAVID, PPI and KEGG analysis showed that the proteins involved in the redox system had significant differences, indicating that the redox state of *A. protothecoides* significantly changed under illumination. Accordingly, the redox properties inside and outside the cells were examined.

**Fig. 6:**
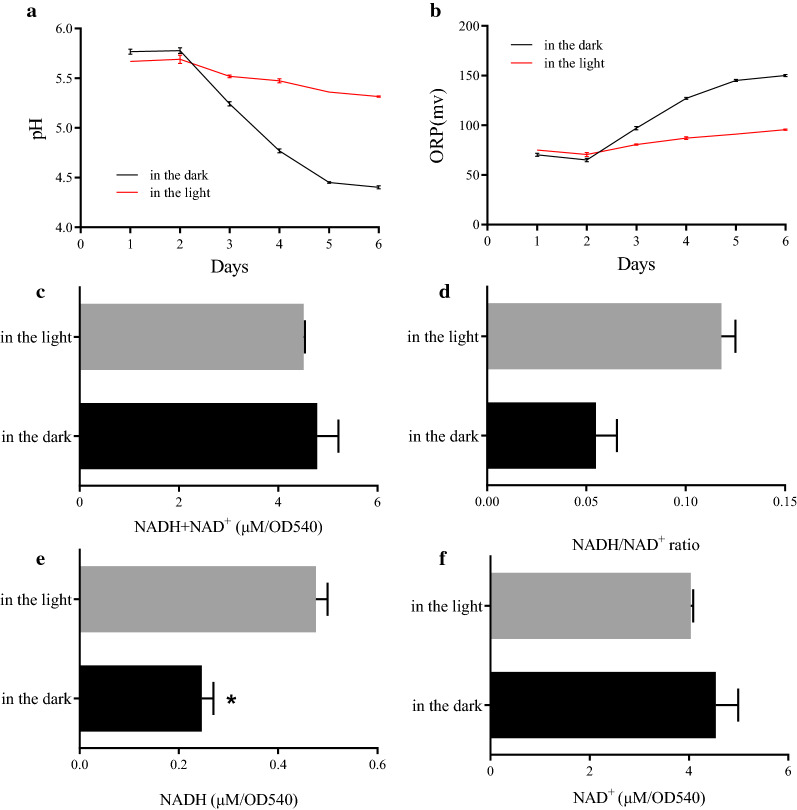
Changes of pH, ORP and NAD^+^/NADH levels under MC and HC conditions. **a** The pH of the medium under the light and dark conditions; **b** the ORP of the medium under the light and dark conditions; **c** total NADH and NAD^+^ pools in the cells grown under the light and dark conditions; **d** NADH/NAD^+^ ratios of cells grown under the light and dark conditions; **e** NADH amounts in the cells grown under the light and dark conditions; **f** NAD^+^ amounts in the cells grown under the light and dark conditions

The pH value and oxidation–reduction potential (ORP) of the culture supernatant in HC and MC were measured using a pH meter. The ORP value reflects the relative degree of oxidative and reductive properties in the medium. A positive and higher ORP value indicates that it is an oxidative environment and has higher oxidative potential, respectively. As shown in Fig. 6, the pH of the HC gradually decreased, indicating that the acidity increased. At the same time, the ORP value gradually increased, indicating that the oxidative potential increased. While the pH of the MC also gradually decreased, its ORP value increased much less, showing that the oxidation potential of MC was not significantly increased. However, the ORP value was positive, indicating that both the HC and MC were in an oxidative environment; whereby, an increase of oxidation in the medium was prevented under light illumination. The improved glucose uptake of *A. protothecoides* in the dark might due to the stronger oxidative properties of the medium, which increased the activity of the glucose transport system.

Next, the intracellular NAD^+^ and NADH levels were measured. The total amount of NAD^+^ and NADH did not change significantly upon illumination (Fig. 6c). However, the ratio of NADH/NAD^+^ and contents of NADH in the MC was higher than in the HC (Fig. 6d, e). These results showed that illumination affects the cellular homeostasis, which ultimately led to a more reductive intracellular environment. The high intracellular reduction state of the MC might reduce the activity of the glucose transport system. This was similar to the sucrose transporter activity in plant systems, which was lower in reductive environments [21, 36]. Further research will be conducted.

## Conclusions

In this study, we found that glucose assimilation in microalgae could be inhibited by light, resulting in a reduction of biomass growth and lipid accumulation. Comparative proteomic analysis revealed that three OEE proteins were significantly upregulated and the thioredoxin system was changed to balance the intracellular redox state. Furthermore, pathway analysis revealed that the G3P produced through the dark reaction of photosynthesis under illumination would reverse the glycolysis pathway and inhibit glucose metabolism. The decrease of glucose transport activity might be related to the more reduced state of the intracellular and external environment. Finally, a theoretical model of how glucose assimilation in *A. protothecoides* is negatively influenced by light was firstly proposed in this study (Additional file 1: Figure S6).

## Methods

### Strain and culture conditions

*Auxenochlorella protothecoides* strain 0710 was obtained from the Culture Collection of Algae at the University of Texas (Austin, TX, USA) and screened for high lipid yield in the Algae Bioenergy Laboratory at Tsinghua University (Beijing, China). *Auxenochlorella protothecoides* strain Al64 was isolated from 0710 as a mutant with autotrophy deficient. The cells were maintained in a basal medium containing (per liter): 0.7-g KH_2_PO_4_, 0.3-g K_2_HPO_4_, 0.3-g MgSO_4_·7H_2_O, 0.3-mg FeSO_4_·7H_2_O, 0.01-mg thiamine, and 1-mL A5 trace mineral solution [12]. Glycine was selected as the nitrogen source in the study since it is more suitable nitrogen source than NH_4_Cl and urea for growth of *A. protothecoides* [10]. The nitrogen-limited medium was the basal medium with the addition of 30-g/L glucose and 0.5-g/L glycine [13]. The sufficient nitrogen medium was basal medium with the addition of 30-g/L glucose and 5-g/L glycine [19]. All media and the cultivation apparatus were sterilized with steam at 112 °C and 0.12 MPa for 30 min.

The cells were grown in 100-mL flasks on a shaker set at 220 rpm and 28 ± 1 °C for all cultivation modes. Mixotrophic culture (MC): cells were maintained in the nitrogen-limited medium under illumination of 2000 lx. Heterotrophic culture (HC): cells were maintained in the nitrogen-limited medium under dark conditions.

### Determination of biomass, chlorophyll and glucose

Samples comprising 1 mL of the microalgal cell suspension were harvested by centrifugation at 8000 rpm for 5 min and the cell pellets were washed twice with distilled water, dried at 70 °C until constant weight, and weighed to determine the dry cell weight. Glucose concentrations were determined using an SBA-40C enzymatic bio-analyzer (Shandong Academy of Sciences, China).

The chlorophyll content was determined by measuring the absorbance of the methanol extracts at 666 nm as described before [38]. The chlorophyll fluorescence was determined as described in the previous report [39], using a PHYTO-PAM Phytoplankton Analyzer (Walz, Germany). The effective quantum yield of PSII (ФPSII) was determined as follows ФPSII = (Fmʹ − Fs)/Fmʹ.

Glucose uptake by cells was assayed as described before [21, 40]. For each assay, 1-μCi d-[2-^3^H] glucose (specific radioactivity 21.5 Ci mmol^−1^; PerkinElmer, USA) was added into 100-μL PBS containing 0.1 OD_540_ of cells. The final concentration of the external d-[2-^3^H] glucose was 0.46 μM. The uptake of radiolabelled substrate was stopped at 180 s by rapidly filtering the solution through a 0.22-μm pore-size membrane (Millipore, USA). The filter membranes were immediately washed with 2-mL ice-cold PBS, solubilized with 500 μL Optiphase HISAFE 3 (PerkinElmer) and used for liquid scintillation counting on a MicroBeta JET instrument (PerkinElmer). Microalgae cells without d-[2-^3^H] glucose were used as a negative control. All counter-flow assays were performed at 25 °C and repeated at least three times. The error bars represent the standard deviations from three replicates.

### Determination of starch, lipid and protein

*A. protothecoides* cells were cultured as described in methodology and collected at 120 h. The total cellular lipid was measured using the procedures described by Jia et al. [41]. The protein has been extracted according to the procedure as previously described by Gao et al. [42]. The concentration of soluble protein was determined by following the instructions of Bradford Protein Assay Kit (C503031, Sangon Biotech, China). A Starch Detection Kit (BC0700, Solarbio, China) was used to determine the starch contents.

### Transmission electron microscopy

*Auxenochlorella protothecoides* cells were pretreated using a standard protocol, including dehydration, embedding and sectioning [14]. The analysis was performed at the microscope facility in the School of Life Sciences of Tsinghua University using a H7650B transmission electron microscope (Hitachi, Japan), as described previously [2].

### Quantitative proteomic analysis by 2D LC–MS/MS

The quantitative proteomic analysis was conducted as described previously [43, 44]. Proteins were extracted from *A. protothecoides* cells cultured for 5 days in MC and HC, respectively, which originally has the density of OD540 = 0.1. The extraction was performed using 8-M urea, and samples comprising 200 μg of total protein were reduced and alkylated. Next, the proteins were digested with trypsin (Promega, Fitchburg, WI) at 37 °C overnight. Tryptic peptides were desalted and labeled with the tandem mass tag (TMT, Thermo Waltham, MA, USA) according to the manufacturer’s protocol. Labeled peptides from different samples were mixed together, desalted and separated using a Dionex UltiMate 3000 two-dimensional HPLC system (Thermo Fisher Scientific, USA). In the first step, reversed-phase separation was performed on an HPLC system at pH 10.0, which yielded 47 fractions that were combined into 12 fractions after rotary evaporation, and then re-suspended in 0.1% formic acid for the next separation. For LC–MS/MS analysis, the TMT-labeled peptides were separated by HPLC at a lower pH via a 120-min gradient elution at a flow rate of 0.250 mL/min. A Q-Extractive mass spectrometer was used in the data-dependent acquisition mode. Each full-scan mass spectrum in the OrbiTrap (300–1800 m/z, 70,000 resolution) was followed by 20 data-dependent MS/MS scans. The MS/MS spectra from each LC–MS/MS run were searched against the UniProt *A. protothecoides* database using the SEQUEST search engine of Proteome Discoverer software. The false discovery rate was estimated and the cutoff score of 1% was accepted based on the decoy database. Relative protein quantification was performed using PD 1.4 software according to the manufacturer’s instructions based on the intensity of six TMT reporter ions per peptide. The analysis was carried out in biological triplicates.

### Bioinformatic analysis

The identified proteins with a Sum PEP Score of more than 5, fold change of more than 1.3 times, and *p*-value of less than 0.05 were considered to be a differentially expressed proteins (DEP). The DAVID website was used to cluster the up- and downregulated proteins [27]. The clustering options include functional categories (UP KEYWORDS), gene ontology (GOTERM BP DIRECT, GOTERM CC DIRECT and GOTERM MF DIRECT) and protein domains (INTERPRO, PIR SUPERFAMILY and SMART).

The up- and downregulated proteins were next uploaded to KEGG Mapper for metabolic pathway enrichment. The upregulated proteins were marked in red, the downregulated protein were marked in blue, and the annotated proteins in *A. protothecoides* the protein database were marked green.

The online protein–protein interaction network database STRING (STRING: functional protein association networks) was used to query protein interactions. The *Chlamydomonas reinhardtii* protein–protein interaction network was used as a reference for protein homology-based analysis of the protein–protein interaction network of *A. protothecoides* based on the differentially expressed proteins with fold changes of more than 1.3 times. Visual interaction analysis of the protein–protein interaction network was performed using Cytoscape software.

### ***Measurements of the intracellular concentrations of NADH and NAD***^+^

*Auxenochlorella protothecoides* cells were harvested during the exponential phase by centrifugation at 3000*g* and 4 °C for 5 min. Then, the cell concentration was adjusted to OD_540_ = 1 using ice-cold PBS (pH 7.4). Samples comprising 300 μL of the normalized cell cultures were collected from each sample, and washed twice with PBS. The concentrations of NAD^+^ (Nicotinamide adenine dinucleotide) and NADH (Nicotinamide adenine dinucleotide reduced form) were measured E2ND-100 NAD^+^/NADH Assay Kit purchased from Bioassay Systems (USA) according to the manufacturer’s instructions.

### Statistical analysis

The results were expressed as the means and standard deviations of triplicate experiments. Statistical analysis was conducted using GraphPad Prism 7.0 software. Student’s *t*-test was used to assess the significance of differences between two sets of data, while multiple comparisons were performed using ANOVA. Differences with *p*-values of < 0.05 were considered statistically significant.

## Supplementary information

**Additional file 1: Figure S1.** TEM images of *A. protothecoides* cells from HC and MC. C: chloroplast; L: lipid bodies; S: starch granules. **(A)** Cells from the illuminated culture (× 1.2 K); **(B)** Cells from the illuminated culture (× 3 K); **(C)** Cells from the dark culture (× 1.2 K); **(D)** Cells from the dark culture (× 3 K). **Figure S2.** Quantum yield (Φ) of PSII of *A. protothecoides* under the light and dark conditions with 0.5-g/L glycine. **Figure S3.** DEPs in photosynthesis. **Figure S4.** DEPs in fatty acid biosynthesis. **Figure S5.** DEPs in citrate cycle (TCA cycle). **Figure S6.** Schematic representation of a theoretical model of how glucose assimilation in *A. protothecoides* is negatively influenced by light.

**Additional file 2: Table S1.** Downregulated proteins of the comparative proteome. **Table S2.** Upregulated proteins of the comparative proteome.

## Data Availability

All data generated or analysed during this study are included in this published article.

## Abbreviations

DEPs: Differentially expressed proteins
FAD: Flavin adenine dinucleotide
G3P: Glyceraldehyde-3-phosphate
HC: Heterotrophic culture
HPLC: High-performance liquid chromatography
LC–MS/MS: Liquid chromatography–tandem mass spectrometry
MC: Mixotrophic culture
OEC: Oxygen-evolving complex
OEE: Oxygen-evolving enhancer proteins
ORP: Oxidation–reduction potential
PBS: Phosphate buffer saline
PSI: Photosystem I
PSII: Photosystem II
PPI: Protein–protein interaction
PUFAs: Polyunsaturated fatty acids
RuBP: Ribulose bisphosphate
TCA: Tricarboxylic acid cycle
TEM: Transmission electron microscope
TMT: Tandem mass tag
VHL: Very high light
