# Supplementary material for: Inhibition of glucose assimilation in Auxenochlorella protothecoides by light
Source: Biotechnol Biofuels. 2020 Aug 18;13:146. doi: 10.1186/s13068-020-01787-9 (PMC7437033; doi:10.1186/s13068-020-01787-9)
Supplement: Supplementary file 1 — Additional file 1: Figure S1. TEM images of A. protothecoides cells from HC and MC. C: chloroplast; L: lipid bodies; S: starch granules. (A) Cells from the illuminated culture (× 1.2 K); (B) Cells from the illuminated culture (× 3 K); (C) Cells from the dark culture (× 1.2 K); (D) Cells from the dark culture (× 3 K). Figure S2. Quantum yield (Φ) of PSII of A. protothecoides under the light and dark conditions with 0.5-g/L glycine. Figure S3. DEPs in photosynthesis. Figure S4. DEPs in fatty acid biosynthesis. Figure S5. DEPs in citrate cycle (TCA cycle). Figure S6. Schematic representation of a theoretical model of how glucose assimilation in A. protothecoides is negatively influenced by light. [file 13068_2020_1787_MOESM1_ESM.docx]

**Additional file**


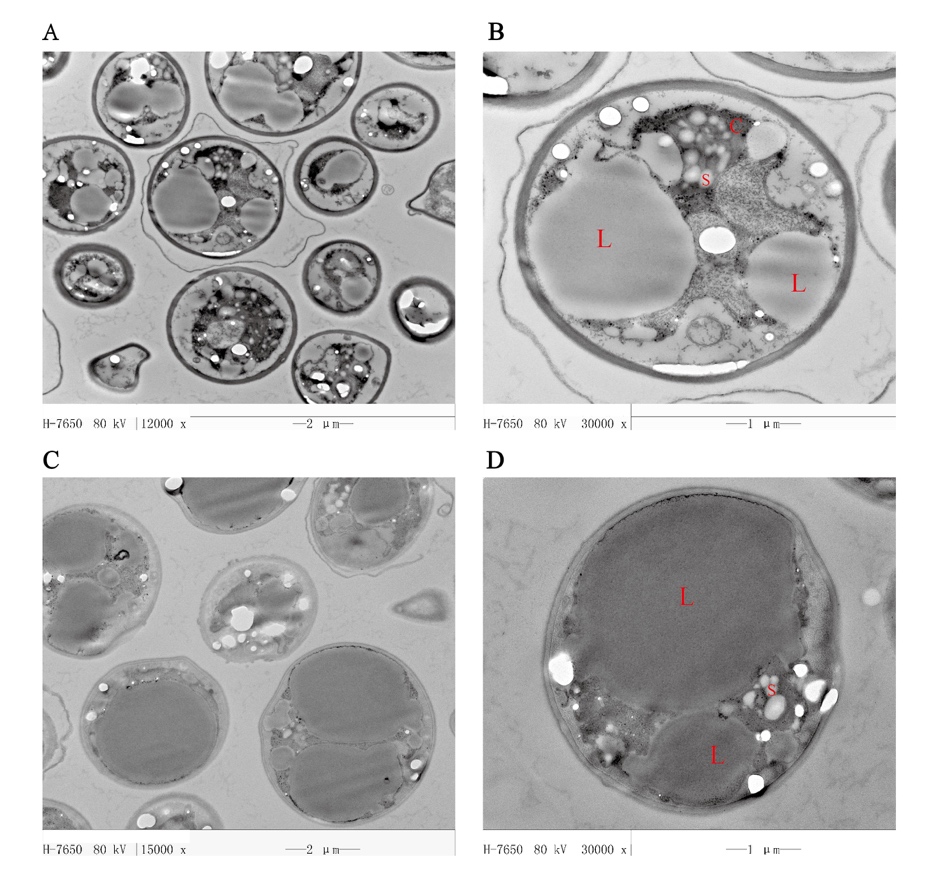


**Figure S1** TEM images of *A. protothecoides* cells from HC and MC. C: chloroplast; L: lipid bodies; S: starch granules. **(A)** Cells from the illuminated culture (×1.2K); **(B)** Cells from the illuminated culture (×3K); **(C)** Cells from the dark culture (×1.2K); **(D)** Cells from the dark culture (×3K).

**Figure S2** Quantum yield (Φ) of PSII of *A. protothecoides* under the light and dark conditions with 0.5 g/L glycine.


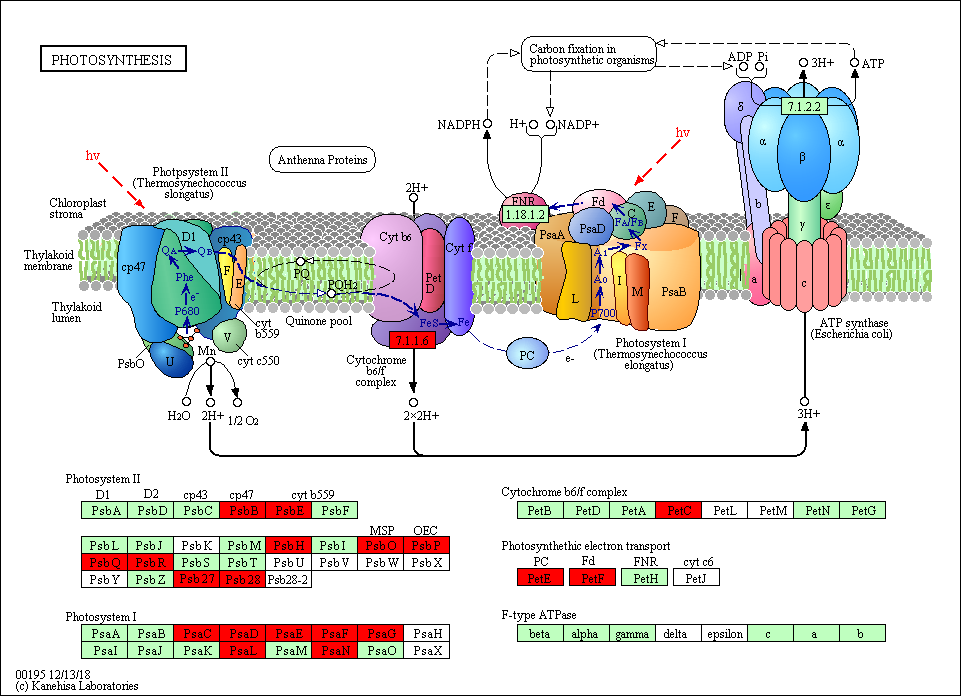


**Figure S3** DEPs in photosynthesis.


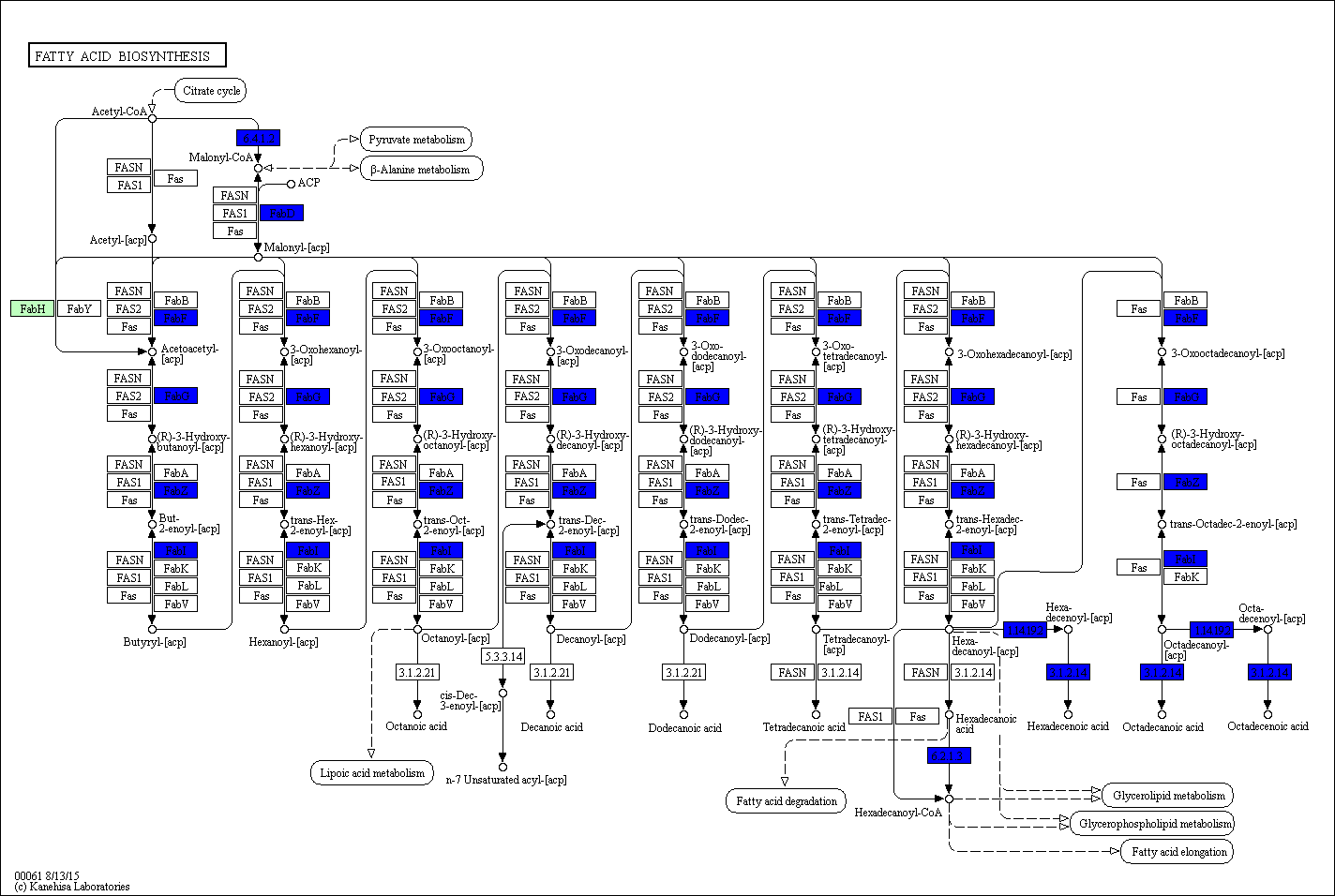


**Figure S4** DEPs in fatty acid biosynthesis.


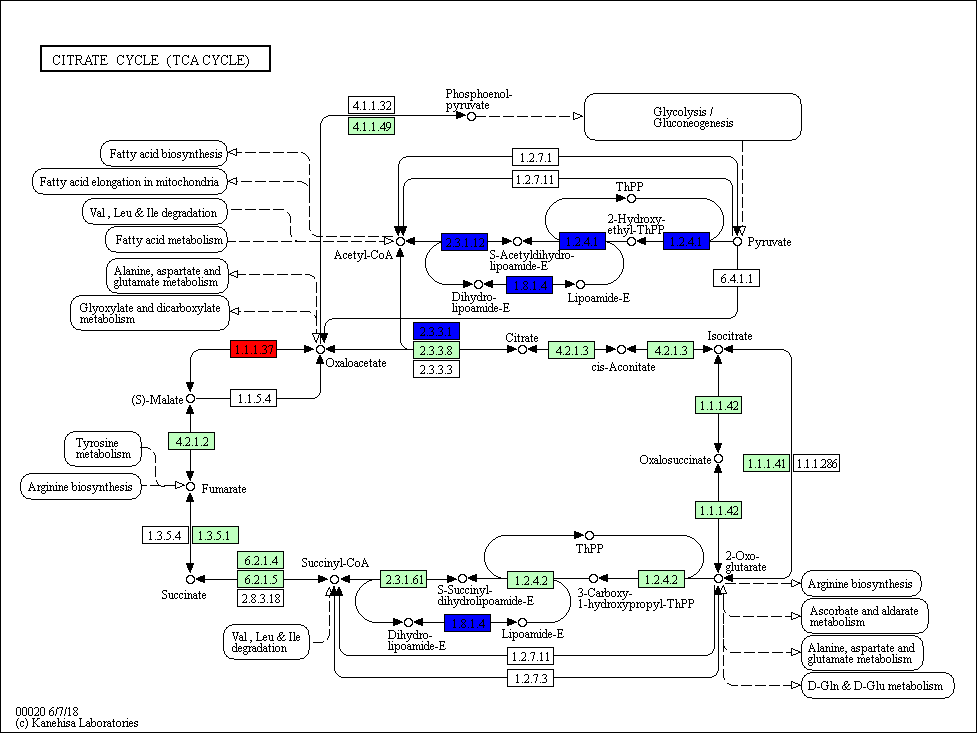


**Figure S5** DEPs in citrate cycle (TCA cycle).


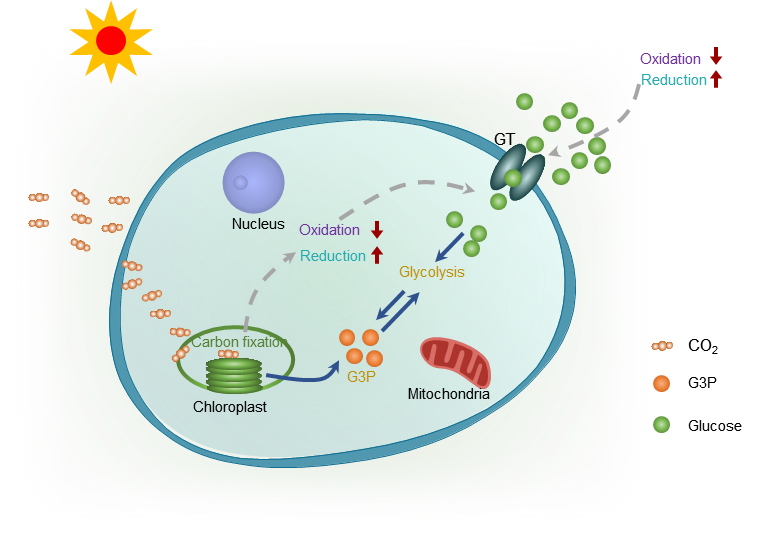


**Figure S6** Schematic representation of a theoretical model of how glucose assimilation in *A. protothecoides* is negatively influenced by light.
