# Supplementary material for: Inhibition of glucose assimilation in Auxenochlorella protothecoides by light
Source: Biotechnol Biofuels. 2020 Aug 18;13:146. doi: 10.1186/s13068-020-01787-9 (PMC7437033; doi:10.1186/s13068-020-01787-9)
Supplement: Supplementary file 2 — Additional file 2: Table S1. Downregulated proteins of the comparative proteome. Table S2. Upregulated proteins of the comparative proteome. [file 13068_2020_1787_MOESM2_ESM.docx]

**Additional tables**

Table S1. Downregulated proteins of the comparative proteome.

| Accession | Description | Avg. fold increase | P value |
| --- | --- | --- | --- |
| A0A087SNS5 | Lipase OS | 0.41 | 0.01 |
| A0A087S9F3 | Uncharacterized protein OS | 0.47 | 0.01 |
| A0A087SHC6 | Long chain acyl-CoA synthetase 7, peroxisomal OS | 0.48 | 0.01 |
| A0A087SCB8 | Uncharacterized protein YciO OS | 0.52 | 0.00 |
| A0A087SI33 | 3-oxoacyl-[acyl-carrier-protein] synthase OS | 0.55 | 0.01 |
| A0A087SJU4 | Biotin carboxyl carrier protein of acetyl-CoA carboxylase, chloroplastic OS | 0.56 | 0.01 |
| A0A087SSY4 | Acyl-[acyl-carrier-protein] hydrolase OS | 0.57 | 0.01 |
| A0A087SLR4 | Putative WD repeat-containing protein OS | 0.57 | 0.00 |
| A0A087SQF9 | Enoyl-[acyl-carrier-protein] reductase [NADH], chloroplastic OS | 0.57 | 0.00 |
| A0A087SD26 | Peptidyl-prolyl cis-trans isomerase cypE OS | 0.58 | 0.00 |
| A0A087S9F3 | Uncharacterized protein OS | 0.59 | 0.02 |
| A0A087SJV3 | Aldehyde dehydrogenase family 2 member B4, mitochondrial (Fragment) OS | 0.59 | 0.01 |
| A0A087SSV9 | Acyl carrier protein OS | 0.60 | 0.03 |
| A0A087SIC7 | L-gulonolactone oxidase OS | 0.60 | 0.00 |
| A0A087SSJ1 | LisH domain-containing protein C29A3.03c (Fragment) OS | 0.60 | 0.04 |
| A0A087SB27 | Acyl-[acyl-carrier-protein] desaturase 5, chloroplastic OS | 0.60 | 0.00 |
| A0A087S9F3 | Uncharacterized protein OS | 0.60 | 0.04 |
| A0A087SCG8 | Coproporphyrinogen-III oxidase, chloroplastic OS | 0.61 | 0.00 |
| A0A087SPL0 | Transaldolase OS | 0.62 | 0.00 |
| A0A087SUD8 | Succinate/fumarate mitochondrial transporter OS | 0.62 | 0.01 |
| A0A087STX1 | 3-hydroxyacyl-[acyl-carrier-protein] dehydratase FabZ OS | 0.63 | 0.00 |
| A0A087SN66 | Biotin carboxylase, chloroplastic OS | 0.63 | 0.00 |
| A0A087SSH0 | Dihydrolipoamide acetyltransferase component of the pyruvate dehydrogenase complex OS | 0.64 | 0.00 |
| A0A087SNR4 | Putative dioxygenase OS | 0.65 | 0.00 |
| A0A087SI08 | 5-methylthioadenosine/S-adenosyl homocysteine deaminase OS | 0.65 | 0.00 |
| A0A087SAZ6 | Biotin carboxyl carrier protein of acetyl-CoA carboxylase OS | 0.65 | 0.00 |
| A0A087SRJ0 | Dihydrolipoyl dehydrogenase OS | 0.66 | 0.00 |
| A0A087S9F3 | Uncharacterized protein OS | 0.66 | 0.02 |
| A0A087SMK8 | Putative malonyl-CoA-acyl carrier protein transacylase, mitochondrial OS | 0.66 | 0.01 |
| A0A087S9F3 | Uncharacterized protein OS | 0.66 | 0.02 |
| A0A087SI38 | Acetyl-coenzyme A synthetase OS | 0.67 | 0.01 |
| A0A087SIE0 | Copper transport protein ATOX1 OS | 0.67 | 0.01 |
| A0A087SKG7 | 4-alpha-glucanotransferase OS | 0.67 | 0.00 |
| A0A087SR35 | Cytochrome b5 isoform B OS | 0.67 | 0.03 |
| A0A087SJW8 | 3-oxoacyl-[acyl-carrier-protein] synthase II, chloroplastic (Fragment) OS | 0.67 | 0.00 |
| A0A087SH81 | Pyruvate dehydrogenase E1 component subunit beta OS | 0.67 | 0.02 |
| A0A087S9F3 | Uncharacterized protein OS | 0.67 | 0.00 |
| A0A087SNS4 | Putative alcohol dehydrogenase OS | 0.68 | 0.01 |
| A0A087S9F3 | Uncharacterized protein OS | 0.68 | 0.00 |
| A0A087SA57 | Isoamylase 3, chloroplastic OS | 0.68 | 0.00 |
| A0A087S9F3 | Uncharacterized protein OS | 0.68 | 0.00 |
| A0A087SH74 | 3-oxoacyl-[acyl-carrier-protein] reductase 3, chloroplastic OS | 0.69 | 0.00 |
| A0A087SMH0 | Pyruvate dehydrogenase E1 component subunit alpha OS | 0.69 | 0.00 |
| A0A087SES6 | Zinc finger CCCH domain-containing protein 24 OS | 0.69 | 0.04 |
| A0A087SFT7 | GTP-binding protein SAR1A OS | 0.69 | 0.01 |
| A0A087SB99 | Putative S-adenosyl-L-methionine-dependent methyltransferase OS | 0.70 | 0.02 |
| A0A087STQ3 | Acyl-coenzyme A oxidase 4, peroxisomal OS | 0.70 | 0.03 |
| A0A087SB27 | Acyl-[acyl-carrier-protein] desaturase 5, chloroplastic OS | 0.70 | 0.02 |
| A0A087SKB1 | Microsomal glutathione S-transferase 3 OS | 0.70 | 0.02 |
| A0A087S9F3 | Uncharacterized protein OS | 0.71 | 0.01 |
| A0A087SU11 | Nudix hydrolase 8 OS | 0.71 | 0.02 |
| A0A087SG29 | Glucose-6-phosphate isomerase OS | 0.71 | 0.01 |
| A0A087SIG7 | Leucine-tRNA ligase OS | 0.71 | 0.01 |
| A0A087S9X9 | LRR receptor-like serine/threonine-protein kinase GSO2 OS | 0.71 | 0.02 |
| A0A087SFJ9 | Glutamate dehydrogenase OS | 0.72 | 0.00 |
| A0A087SCX1 | Phosphoglucan, water dikinase, chloroplastic OS | 0.72 | 0.00 |
| A0A087SGI7 | Plasma membrane calcium-transporting ATPase 3 OS | 0.72 | 0.04 |
| A0A087STB9 | Acyl-coenzyme A oxidase OS | 0.72 | 0.00 |
| A0A087SPH9 | 3-ketoacyl-CoA thiolase 2, peroxisomal OS | 0.72 | 0.03 |
| A0A087S9U0 | Acetyl-coenzyme A carboxylase carboxyl transferase subunit beta, chloroplastic (Fragment) OS | 0.72 | 0.03 |
| A0A087S9F3 | Uncharacterized protein OS | 0.73 | 0.02 |
| A0A087SNJ5 | ADP-ribosylation factor-like protein 2 OS | 0.73 | 0.02 |
| A0A087SA62 | Putative plastid-lipid-associated protein 8, chloroplastic OS | 0.73 | 0.05 |
| A0A087SJJ2 | Ubiquinol oxidase OS | 0.73 | 0.05 |
| A0A087SQ73 | Serine/threonine-protein phosphatase 1 OS | 0.73 | 0.00 |
| A0A087SGD4 | D-aspartate oxidase OS | 0.73 | 0.02 |
| A0A087SBG4 | UPF0420 protein C16orf58-like protein OS | 0.73 | 0.00 |
| A0A087SN14 | Pyruvate kinase OS | 0.73 | 0.02 |
| A0A087S9F3 | Uncharacterized protein OS | 0.74 | 0.01 |
| A0A087S9F3 | Uncharacterized protein OS | 0.74 | 0.03 |
| A0A087SN14 | Pyruvate kinase OS | 0.74 | 0.00 |
| A0A087S9F3 | Uncharacterized protein OS | 0.74 | 0.01 |
| A0A087SBU0 | B3 domain-containing protein VP1 OS | 0.74 | 0.01 |
| A0A087SD72 | 3-deoxy-manno-octulosonate cytidylyltransferase OS | 0.74 | 0.03 |
| A0A087SPN9 | Proline iminopeptidase OS | 0.75 | 0.00 |
| A0A087SJV6 | Glycerol-3-phosphate dehydrogenase [NAD^+^] OS | 0.75 | 0.04 |
| A0A087ST50 | KDEL-tailed cysteine endopeptidase CEP1 OS | 0.75 | 0.03 |
| A0A087S9F3 | Uncharacterized protein OS | 0.75 | 0.03 |
| A0A087SLZ7 | Citrate synthase OS | 0.75 | 0.03 |
| A0A087SN14 | Pyruvate kinase OS | 0.76 | 0.01 |
| A0A087S9F3 | Uncharacterized protein OS | 0.76 | 0.01 |
| A0A087SL07 | Phosphate acetyltransferase OS | 0.76 | 0.01 |
| A0A087SRR1 | Amine oxidase OS | 0.76 | 0.02 |
| A0A087SQ39 | BEACH domain-containing protein LvsF OS | 0.76 | 0.00 |
| A0A087SCK3 | Fructose-bisphosphate aldolase OS | 0.76 | 0.00 |
| A0A087SEW0 | Alpha-1,4 glucan phosphorylase OS | 0.76 | 0.00 |
| A0A087SDB4 | Ketol-acid reductoisomerase, chloroplastic OS | 0.76 | 0.00 |
| A0A087S9F3 | Uncharacterized protein OS | 0.76 | 0.05 |
| A0A087SB76 | Agmatine deiminase OS | 0.76 | 0.02 |
| A0A087SLY9 | Malate synthase OS | 0.76 | 0.02 |
| A0A087SN14 | Pyruvate kinase OS | 0.76 | 0.01 |
| A0A087SGR9 | Serine/threonine-protein kinase Nek1 OS | 0.76 | 0.01 |
| A0A087SBQ4 | Protein NLRC3 OS | 0.76 | 0.01 |
| A0A087STT2 | Nuclear polyadenylated RNA-binding protein 4 (Fragment) OS | 0.77 | 0.02 |
| A0A087SM91 | Cytochrome c peroxidase, mitochondrial OS | 0.77 | 0.02 |
| A0A087SC55 | Phospholipase DDHD1 OS | 0.77 | 0.00 |
| A0A087S9F3 | Uncharacterized protein OS | 0.77 | 0.03 |

Table S2. Upregulated proteins of the comparative proteome.

| Accession | Description | Avg. fold increase | P value |
| --- | --- | --- | --- |
| A0A087SNB8 | Chlorophyll a-b binding protein, chloroplastic OS | 10.68 | 0.00 |
| A0A087SJP2 | Photosystem I reaction center subunit N, chloroplastic (Fragment) OS | 9.06 | 0.00 |
| A0A087SBB6 | Photosystem I reaction center subunit V, chloroplastic OS | 8.36 | 0.00 |
| A0A087SNJ7 | Oxygen-evolving enhancer protein 1, chloroplastic OS | 8.09 | 0.00 |
| A0A087SK18 | Photosystem I reaction center subunit XI, chloroplastic OS | 7.03 | 0.00 |
| A0A087SC76 | Oxygen-evolving enhancer protein 2, chloroplastic OS | 6.96 | 0.00 |
| A0A087SP25 | Oxygen-evolving enhancer protein 3, chloroplastic OS | 6.21 | 0.00 |
| A0A087SNZ6 | Photosystem II 10 kDa polypeptide, chloroplastic OS | 6.19 | 0.00 |
| A0A087SNB8 | Chlorophyll a-b binding protein, chloroplastic OS | 6.07 | 0.00 |
| A0A087S9F3 | Uncharacterized protein OS | 6.07 | 0.00 |
| A0A087SNB8 | Chlorophyll a-b binding protein, chloroplastic OS | 5.37 | 0.00 |
| A0A087SG73 | Photosystem I reaction center subunit III, chloroplastic OS | 5.37 | 0.00 |
| A0A087SNB8 | Chlorophyll a-b binding protein, chloroplastic OS | 5.24 | 0.00 |
| A0A087SEV8 | Photosystem I reaction center subunit IV, chloroplastic OS | 5.16 | 0.00 |
| A0A087SNB8 | Chlorophyll a-b binding protein, chloroplastic OS | 5.15 | 0.00 |
| A0A087S9F3 | Uncharacterized protein OS | 5.05 | 0.00 |
| A0A087SNB8 | Chlorophyll a-b binding protein, chloroplastic OS | 4.98 | 0.00 |
| A0A087S9F3 | Uncharacterized protein OS | 4.94 | 0.00 |
| A0A023HHU3 | Photosystem I iron-sulfur center OS | 4.87 | 0.00 |
| A0A087S9U1 | Cytochrome b559 subunit alpha OS | 4.72 | 0.00 |
| A0A087STY2 | 18.1 kDa class I heat-shock protein OS | 4.57 | 0.00 |
| A0A087S9F3 | Uncharacterized protein OS | 4.42 | 0.00 |
| A0A087SFC6 | Plastocyanin OS | 4.31 | 0.00 |
| A0A087SP85 | Photosystem I reaction center subunit II, chloroplastic OS | 4.20 | 0.00 |
| A0A087SNB8 | Chlorophyll a-b binding protein, chloroplastic OS | 4.17 | 0.00 |
| A0A087S9U7 | Photosystem II reaction center protein H (Fragment) OS | 4.06 | 0.00 |
| A0A087S9F3 | Uncharacterized protein OS | 3.94 | 0.00 |
| A0A087SRZ8 | Photosystem II repair protein PSB27-H1, chloroplastic OS | 3.90 | 0.00 |
| A0A087SM57 | Phosphoribulokinase OS | 3.56 | 0.00 |
| A0A087SCS0 | Magnesium-protoporphyrin O-methyltransferase OS | 3.46 | 0.00 |
| A0A087SIH0 | Geranylgeranyl diphosphate reductase, chloroplastic OS | 3.42 | 0.00 |
| A0A087S9F3 | Uncharacterized protein OS | 3.13 | 0.00 |
| A0A087S9U5 | Photosystem II CP47 chlorophyll apoprotein (Fragment) OS | 3.12 | 0.00 |
| A0A087SAA7 | Serine/threonine-protein kinase STN8, chloroplastic OS | 3.12 | 0.00 |
| A0A087SBC4 | Thylakoid lumen 17.4 kDa protein, chloroplastic OS | 2.99 | 0.00 |
| A0A087SNB8 | Chlorophyll a-b binding protein, chloroplastic OS | 2.97 | 0.01 |
| A0A087SCK9 | Photosystem II stability/assembly factor HCF136, chloroplastic OS | 2.86 | 0.00 |
| A0A087S9F3 | Uncharacterized protein OS | 2.79 | 0.00 |
| A0A087SQ10 | Ribulose bisphosphate carboxylase/oxygenase activase, chloroplastic OS | 2.78 | 0.00 |
| A0A087STT4 | Thylakoid lumen protein, chloroplastic OS | 2.71 | 0.00 |
| A0A087S9F3 | Uncharacterized protein OS | 2.71 | 0.00 |
| A0A087SCR0 | Uncharacterized protein ycf39 OS | 2.65 | 0.00 |
| A0A087SKJ3 | Glyceraldehyde-3-phosphate dehydrogenase OS | 2.62 | 0.00 |
| A0A087SAW7 | Ribulose bisphosphate carboxylase small chain OS | 2.58 | 0.00 |
| A0A087SQQ7 | Cytochrome b6-f complex iron-sulfur subunit OS | 2.52 | 0.00 |
| A0A087SMZ0 | Photosystem II reaction center Psb28 protein OS | 2.38 | 0.00 |
| A0A087S9F3 | Uncharacterized protein OS | 2.36 | 0.00 |
| A0A087SHS3 | PsbP-like protein 1, chloroplastic OS | 2.34 | 0.00 |
| A0A087S9F3 | Uncharacterized protein OS | 2.30 | 0.01 |
| A0A087S9K2 | Ferredoxin OS | 2.21 | 0.00 |
| A0A087S9F3 | Uncharacterized protein OS | 2.17 | 0.02 |
| A0A087S9F3 | Uncharacterized protein OS | 2.15 | 0.01 |
| A0A087SS65 | PsbP domain-containing protein 3, chloroplastic OS | 2.12 | 0.01 |
| A0A087SMW7 | Peptidylprolyl isomerase OS | 2.12 | 0.00 |
| A0A087SJB8 | Protein YIPF6 OS | 2.06 | 0.00 |
| A0A087S9F3 | Uncharacterized protein OS | 2.05 | 0.00 |
| A0A087S9F3 | Uncharacterized protein OS | 2.05 | 0.03 |
| A0A087SQC6 | Protein involved in proton gradient regulation 5, chloroplastic (Fragment) OS | 2.02 | 0.00 |
| A0A023HHV0 | Photosystem I assembly protein Ycf3 OS | 2.00 | 0.01 |
| A0A087SB74 | Peptidylprolyl cis-trans isomerase, chloroplastic OS | 1.96 | 0.00 |
| A0A087SIK8 | Serine/threonine-protein kinase stt7, chloroplastic OS | 1.96 | 0.00 |
| A0A087SCR0 | Uncharacterized protein ycf39 OS | 1.92 | 0.00 |
| A0A087SJ17 | Calcium sensing receptor, chloroplastic OS | 1.91 | 0.00 |
| A0A087S9F3 | Uncharacterized protein OS | 1.89 | 0.00 |
| A0A087SRE0 | Putative plastid-lipid-associated protein 6, chloroplastic OS | 1.89 | 0.00 |
| A0A087SLX8 | Protease Do-like 1, chloroplastic OS | 1.87 | 0.00 |
| A0A087S9F3 | Uncharacterized protein OS | 1.87 | 0.01 |
| A0A087S9F3 | Uncharacterized protein OS | 1.83 | 0.01 |
| A0A087S9F3 | Uncharacterized protein OS | 1.82 | 0.00 |
| A0A087SKW8 | Sedoheptulose-1,7-bisphosphatase, chloroplastic OS | 1.82 | 0.00 |
| A0A087SB69 | Uncharacterized protein, chloroplastic OS | 1.82 | 0.00 |
| A0A087SKK7 | Serine-glyoxylate aminotransferase OS | 1.80 | 0.00 |
| A0A087SI46 | Chloroplast stem-loop binding protein of 41 kDa b, chloroplastic OS | 1.79 | 0.00 |
| A0A087S9F3 | Uncharacterized protein OS | 1.79 | 0.00 |
| A0A087S9F3 | Uncharacterized protein OS | 1.77 | 0.03 |
| A0A087S9F3 | Uncharacterized protein OS | 1.76 | 0.03 |
| A0A087SJW1 | PGR5-like protein 1B, chloroplastic OS | 1.75 | 0.01 |
| A0A087SCK3 | Fructose-bisphosphate aldolase OS | 1.72 | 0.00 |
| A0A087S9F3 | Uncharacterized protein OS | 1.72 | 0.00 |
| A0A087SBG8 | Chloroplast stem-loop binding protein of 41 kDa a, chloroplastic OS | 1.71 | 0.00 |
| A0A087SN62 | TBC1 domain family member 2A OS | 1.70 | 0.00 |
| A0A087SMW7 | Peptidylprolyl isomerase OS | 1.68 | 0.00 |
| A0A087SL69 | 6-phosphofructokinase 5, chloroplastic OS | 1.66 | 0.02 |
| A0A087S9F3 | Uncharacterized protein OS | 1.66 | 0.01 |
| A0A087STJ2 | LETM1 and EF-hand domain-containing protein anon-60Da, mitochondrial OS | 1.65 | 0.01 |
| A0A087S9F3 | Uncharacterized protein OS | 1.65 | 0.01 |
| A0A087SN69 | 50S ribosomal protein L10 OS | 1.65 | 0.00 |
| A0A087SMW7 | Peptidylprolyl isomerase OS | 1.64 | 0.01 |
| A0A087SIR7 | Fructose-1,6-bisphosphatase, chloroplastic OS | 1.63 | 0.00 |
| A0A087S9F3 | Uncharacterized protein OS | 1.61 | 0.00 |
| A0A087S9F3 | Uncharacterized protein OS | 1.60 | 0.00 |
| A0A087S9F3 | Uncharacterized protein OS | 1.59 | 0.00 |
| A0A087S9F3 | Uncharacterized protein OS | 1.59 | 0.00 |
| A0A087S9F3 | Uncharacterized protein OS | 1.58 | 0.01 |
| A0A087S9F3 | Uncharacterized protein OS | 1.58 | 0.01 |
| A0A087S9F3 | Uncharacterized protein OS | 1.58 | 0.00 |
| A0A087S9F3 | Uncharacterized protein OS | 1.57 | 0.01 |
| A0A087S9F3 | Uncharacterized protein OS | 1.57 | 0.03 |
| A0A087SI87 | UPF0603 protein, chloroplastic OS | 1.56 | 0.01 |
| A0A087SMT3 | Thioredoxin M-type, chloroplastic OS | 1.56 | 0.00 |
| A0A087SB83 | Acyl-coenzyme A thioesterase THEM4 OS | 1.56 | 0.01 |
| A0A087S9F3 | Uncharacterized protein OS | 1.56 | 0.02 |
| A0A087SLR6 | Uncharacterized protein YhcW OS | 1.55 | 0.02 |
| A0A087SJK1 | Zinc transporter 5 OS | 1.55 | 0.00 |
| A0A087S9F3 | Uncharacterized protein OS | 1.55 | 0.01 |
| A0A087SU41 | Ribulose-phosphate 3-epimerase, chloroplastic OS | 1.55 | 0.01 |
| A0A087SDF1 | Thylakoid lumen 29 kDa protein, chloroplastic OS | 1.54 | 0.02 |
| A0A087S9F3 | Uncharacterized protein OS | 1.54 | 0.05 |
| A0A087SGB5 | Polyribonucleotide 5'-hydroxyl-kinase Clp1 OS | 1.54 | 0.01 |
| A0A087SB69 | Uncharacterized protein, chloroplastic OS | 1.53 | 0.01 |
| A0A087S9F3 | Uncharacterized protein OS | 1.52 | 0.01 |
| A0A087SIQ5 | 30S ribosomal protein S1, chloroplastic OS | 1.52 | 0.00 |
| A0A087S9F3 | Uncharacterized protein OS | 1.51 | 0.01 |
| A0A087STM7 | Protein phosphatase 2C 57 OS | 1.51 | 0.01 |
| A0A087SHX5 | Elongation factor G, mitochondrial OS | 1.50 | 0.00 |
| A0A087S9F3 | Uncharacterized protein OS | 1.49 | 0.00 |
| A0A087SFW9 | Uncharacterized protein YhiN OS | 1.49 | 0.00 |
| A0A087SM18 | Signal recognition particle receptor FtsY OS | 1.49 | 0.02 |
| A0A087SAT6 | Putative oxidoreductase, chloroplastic OS | 1.48 | 0.03 |
| A0A087SH59 | 30S ribosomal protein S6 OS | 1.48 | 0.03 |
| A0A087SD88 | Zeta-carotene desaturase OS | 1.48 | 0.00 |
| A0A087SM25 | Ribosomal protein OS | 1.47 | 0.00 |
| A0A087S9F3 | Uncharacterized protein OS | 1.47 | 0.00 |
| A0A087SQJ4 | Uncharacterized protein (Fragment) OS | 1.46 | 0.00 |
| A0A087SUE0 | Putative low molecular weight protein-tyrosine-phosphatase OS | 1.46 | 0.00 |
| A0A087SD75 | Putative aarF domain-containing protein kinase, chloroplastic OS | 1.46 | 0.03 |
| A0A087SPB9 | ATPase family AAA domain-containing protein 2 OS | 1.46 | 0.03 |
| A0A087SGB0 | Putative oxidoreductase OS | 1.46 | 0.01 |
| A0A087SDC6 | Putative kinetochore protein NUF2 OS | 1.45 | 0.03 |
| A0A087S9F3 | Uncharacterized protein OS | 1.45 | 0.01 |
| A0A087S9J7 | 50S ribosomal protein L6, chloroplastic OS | 1.44 | 0.02 |
| A0A087S9F3 | Uncharacterized protein OS | 1.44 | 0.01 |
| A0A087SBT5 | Transmembrane protein 208-like protein OS | 1.44 | 0.04 |
| A0A087S9S7 | 50S ribosomal protein L31, chloroplastic OS | 1.44 | 0.00 |
| A0A087SEA7 | Malate dehydrogenase OS | 1.44 | 0.01 |
| A0A087SBS9 | Selenium-binding protein 1 OS | 1.43 | 0.00 |
| A0A087SRD6 | Phosphatidate phosphatase PPAPDC1B OS | 1.43 | 0.02 |
| A0A087SS53 | Peroxiredoxin-2 OS | 1.43 | 0.01 |
| A0A087SDT2 | Thioredoxin-like protein CDSP32, chloroplastic OS | 1.43 | 0.01 |
| A0A087S9F3 | Uncharacterized protein OS | 1.43 | 0.01 |
| A0A087S9F3 | Uncharacterized protein OS | 1.43 | 0.00 |
| A0A087S9F3 | Uncharacterized protein OS | 1.42 | 0.03 |
| A0A087SJI6 | 50S ribosomal protein L17, chloroplastic OS | 1.42 | 0.00 |
| A0A087SI99 | 15-cis-phytoene desaturase, chloroplastic/chromoplastic OS | 1.42 | 0.00 |
| A0A087SD81 | Condensin complex subunit 2 OS | 1.41 | 0.02 |
| A0A087SR46 | 50S ribosomal protein L4 OS | 1.41 | 0.00 |
| A0A087S9F3 | Uncharacterized protein OS | 1.41 | 0.00 |
| A0A087SPI0 | 50S ribosomal protein L9 OS | 1.41 | 0.02 |
| A0A087S9F3 | Uncharacterized protein OS | 1.40 | 0.01 |
| A0A087S9F3 | Uncharacterized protein OS | 1.40 | 0.01 |
| A0A087SJD4 | DNA topoisomerase 2 OS | 1.40 | 0.01 |
| A0A087SIN7 | Protein translocase subunit SecA OS | 1.40 | 0.00 |
| A0A087S9F3 | Uncharacterized protein OS | 1.40 | 0.01 |
| A0A087SB69 | Uncharacterized protein, chloroplastic OS | 1.40 | 0.03 |
| A0A087SMJ2 | ABC transporter C family member 5 OS | 1.40 | 0.02 |
| A0A087SNT1 | 30S ribosomal protein S5, chloroplastic OS | 1.39 | 0.00 |
| A0A087S9F3 | Uncharacterized protein OS | 1.39 | 0.03 |
| A0A087SJ54 | Chloroplast processing peptidase OS | 1.39 | 0.00 |
| A0A087S9F3 | Uncharacterized protein OS | 1.39 | 0.00 |
| A0A087S9F3 | Uncharacterized protein OS | 1.39 | 0.04 |
| A0A087S9F3 | Uncharacterized protein OS | 1.39 | 0.01 |
| A0A087SAF1 | Mrp-like protein OS | 1.39 | 0.02 |
| A0A087SEA6 | Transcription factor OS | 1.38 | 0.03 |
| A0A087S9F3 | Uncharacterized protein OS | 1.38 | 0.00 |
| A0A087SDM4 | Chaperone protein DnaJ OS | 1.38 | 0.04 |
| A0A087SE32 | Molybdenum cofactor sulfurtransferase OS | 1.38 | 0.02 |
| A0A087SNZ1 | Peptide methionine sulfoxide reductase A4, chloroplastic OS | 1.38 | 0.02 |
| A0A087STZ2 | Uncharacterized protein ycf53 OS | 1.38 | 0.01 |
| A0A087S9F3 | Uncharacterized protein OS | 1.38 | 0.02 |
| A0A087S9F3 | Uncharacterized protein OS | 1.38 | 0.03 |
| A0A087SBE7 | Serine/threonine-protein kinase D OS | 1.37 | 0.02 |
| A0A087S9F3 | Uncharacterized protein OS | 1.37 | 0.05 |
| A0A087SHH3 | Mitochondrial import inner membrane translocase subunit Tim13-B OS | 1.37 | 0.00 |
| A0A087S9F3 | Uncharacterized protein OS | 1.37 | 0.00 |
| A0A087S9F3 | Uncharacterized protein OS | 1.37 | 0.02 |
| A0A087SLU2 | 17.6 kDa class I heat shock protein 3 OS | 1.37 | 0.00 |
| A0A087S9F3 | Uncharacterized protein OS | 1.37 | 0.01 |
| A0A087SQK8 | Endoglucanase 1 OS | 1.37 | 0.00 |
| A0A087S9F3 | Uncharacterized protein OS | 1.36 | 0.04 |
| A0A087SKH4 | Putative histone chaperone ASF1A OS | 1.36 | 0.01 |
| A0A087SCL1 | Porphobilinogen deaminase, chloroplastic OS | 1.36 | 0.01 |
| A0A087S9F3 | Uncharacterized protein OS | 1.36 | 0.01 |
| A0A087SB69 | Uncharacterized protein, chloroplastic OS | 1.36 | 0.01 |
| A0A087SU02 | 50S ribosomal protein L28 OS | 1.36 | 0.01 |
| A0A087SNN6 | Stress-induced phosphoprotein 1 OS | 1.36 | 0.00 |
| A0A087SCI5 | Elongation factor Ts (Fragment) OS | 1.35 | 0.03 |
| A0A087SKH8 | 10 kDa chaperonin OS | 1.35 | 0.01 |
| A0A087SKK4 | 30S ribosomal protein S1 OS | 1.35 | 0.02 |
| A0A087SRS9 | 30S ribosomal protein S10, chloroplastic OS | 1.35 | 0.04 |
| A0A087S9S6 | Kinesin-like protein (Fragment) OS | 1.35 | 0.02 |
| A0A087SB98 | Mannan endo-1,4-beta-mannosidase 1 OS | 1.35 | 0.02 |
| A0A087SMT0 | 50S ribosomal protein L29 OS | 1.35 | 0.00 |
| A0A087SCT3 | 50S ribosomal protein L24 OS | 1.35 | 0.02 |
| A0A087SSB4 | 50S ribosomal protein L13, chloroplastic OS | 1.35 | 0.01 |
| A0A087SDH1 | Cyclin-dependent kinase B1-1 OS | 1.35 | 0.05 |
| A0A087SF36 | Ferredoxin-1 OS | 1.35 | 0.00 |
| A0A087SJJ6 | Mitochondrial import inner membrane translocase subunit Tim8 A OS | 1.35 | 0.00 |
| A0A087S9F3 | Uncharacterized protein OS | 1.34 | 0.00 |
| A0A087SC10 | DNA-directed RNA polymerases I, II, and III subunit RPABC2 OS | 1.34 | 0.01 |
| A0A087SKP8 | Putative signal recognition particle 43 kDa protein, chloroplastic OS | 1.34 | 0.02 |
| A0A087SC99 | Replication factor C subunit 2 OS | 1.34 | 0.01 |
| A0A087S9F3 | Uncharacterized protein OS | 1.34 | 0.01 |
| A0A087SNE8 | Seed maturation protein PM36 OS | 1.34 | 0.01 |
| A0A087SMB3 | 50S ribosomal protein L21, chloroplastic OS | 1.33 | 0.00 |
| A0A087SD75 | Putative aarF domain-containing protein kinase, chloroplastic OS | 1.33 | 0.04 |
| A0A087SBI6 | Deoxyribodipyrimidine photo-lyase OS | 1.33 | 0.04 |
| A0A087S9F3 | Uncharacterized protein OS | 1.33 | 0.02 |
| A0A087ST98 | Putative transcription-associated protein 1 OS | 1.33 | 0.01 |
| A0A087STY9 | Endoplasmin-like protein OS | 1.33 | 0.00 |
| A0A087SKC5 | Ferredoxin-thioredoxin reductase catalytic chain, chloroplastic OS | 1.33 | 0.01 |
| A0A087SS88 | Gamma-glutamyl hydrolase A OS | 1.33 | 0.03 |
| A0A087SS95 | Proliferating cell nuclear antigen OS | 1.33 | 0.02 |
| A0A087SE64 | Heat shock protein 83-1 OS | 1.32 | 0.00 |
| A0A087S9F3 | Uncharacterized protein OS | 1.32 | 0.00 |
| A0A087SMW7 | Peptidylprolyl isomerase OS | 1.32 | 0.01 |
| A0A087STE0 | Desumoylating isopeptidase 1 OS | 1.32 | 0.01 |
| A0A087S9F3 | Uncharacterized protein OS | 1.32 | 0.03 |
| A0A087SKE7 | Glutaredoxin-C4 OS | 1.32 | 0.00 |
| A0A087SBA0 | Desiccation-related protein PCC13-62 OS | 1.32 | 0.03 |
| A0A087S9F3 | Uncharacterized protein OS | 1.31 | 0.00 |
| A0A087S9F3 | Uncharacterized protein OS | 1.31 | 0.03 |
| A0A087SKH8 | 10 kDa chaperonin OS | 1.31 | 0.01 |
| A0A087SAP8 | Hydroxysteroid 11-beta-dehydrogenase 1-like protein OS | 1.31 | 0.02 |
| A0A087S9F3 | Uncharacterized protein OS | 1.31 | 0.03 |
| A0A087SN01 | Putative oxidoreductase C1F5.03c OS | 1.31 | 0.02 |
| A0A087SEI1 | Putative plastid-lipid-associated protein 4, chloroplastic (Fragment) OS | 1.31 | 0.00 |
| A0A087SQY7 | Glucomannan 4-beta-mannosyltransferase 9 OS | 1.31 | 0.00 |
| A0A087SMP3 | Putative RING finger protein OS | 1.31 | 0.00 |
| A0A087SJK8 | Protein involved in thylakoid formation 1, chloroplastic OS | 1.31 | 0.00 |
| A0A087SIY7 | Major facilitator superfamily domain-containing protein 5 OS | 1.31 | 0.03 |
| A0A087S9F3 | Uncharacterized protein OS | 1.31 | 0.01 |
| A0A087SU27 | Calcium-dependent protein kinase 11 OS | 1.30 | 0.04 |
| A0A087SDY7 | 50S ribosomal protein L15, chloroplastic OS | 1.30 | 0.01 |
| A0A087STC1 | Magnesium-chelatase subunit H OS | 1.30 | 0.04 |
| A0A087SCD4 | GDP-mannose 3,5-epimerase 2 OS | 1.30 | 0.00 |
| A0A087SCN4 | Thioredoxin reductase OS | 1.30 | 0.01 |
| A0A087SJM2 | CBS domain-containing protein CBSX2, chloroplastic OS | 1.30 | 0.01 |
| A0A087SEB0 | Endoglucanase B OS | 1.30 | 0.00 |
| A0A087SLS2 | GTP-binding protein TypA/BipA-like protein OS | 1.30 | 0.02 |
